# Different approaches to characterize artificial breeding sites of *Aedes aegypti* using generalized linear mixed models

**DOI:** 10.1186/s40249-020-00705-3

**Published:** 2020-07-31

**Authors:** Nicolás Flaibani, Adriana A. Pérez, Ignacio M. Barbero, Nora E. Burroni

**Affiliations:** 1 Department of Ecology, Genetics and Evolution, Faculty of Exact and Natural Sciences, University of Buenos Aires, Buenos Aires, Argentina; 2 Faculty of Exact and Natural Sciences, Institute of Ecology, Genetics and Evolution of Buenos Aires (IEGEBA-CONICET), University of Buenos Aires-National Council for Scientific and Technical Research, Buenos Aires, Argentina; 3 Applied Biostatistics Group, Department of Ecology, Genetics and Evolution, Faculty of Exact and Natural Sciences, University of Buenos Aires, Buenos Aires, Argentina; 4 Study Group of Mosquitoes, Department of Ecology, Genetics and Evolution, Faculty of Exact and Natural Sciences, University of Buenos Aires, Buenos Aires, Argentina; 5 Laboratory of Study of Insect Biology, Center for Scientific Research and Technology Transfer to Production (CICyTTP-CONICET-Entre Ríos-UADER), Diamante, Entre Ríos Argentina

**Keywords:** Containers, Immature mosquitoes, Vectors

## Abstract

**Background:**

As no globally accepted dengue vaccines or specific antiviral therapies are currently available, controlling breeding sites of *Aedes aegypti* is a target to prevent dengue outbreaks. The present study aimed to characterize outdoor artificial breeding sites in urban households using an exhaustive classification system.

**Methods:**

A cross-sectional entomological survey was carried out in Colón city, Entre Ríos, Argentina, using a two-stage stratified sampling design during March and April 2014. The city was stratified given the degree of urbanization of each block, and blocks and households were randomly selected. All outdoor containers with water were inspected, and the presence of immature mosquitoes was recorded. Containers were classified according to physical, functional, and location attributes. Generalized linear mixed models were applied to take into account the aggregated nature of the data (containers in houses and houses in blocks).

**Results:**

Overall, 207 houses were inspected. Out of 522 containers with water, 25% had immatures of *Ae. aegypti* (7336). In adjusted models, the abundance of immatures was higher in containers with increasing opening surface and volume, without roof cover, exposed to shadow, out of use or with functions related to gardening activities, household chores, water storage, or construction. At block level, immatures abundance was positively associated with the degree of urbanization.

**Conclusions:**

We detected high immatures abundance in containers associated with water utilization. This suggests that containers involved in these activities, whether directly (e.g., water storage) or indirectly (e.g., incomplete water drainage in the last use), are susceptible to present a high immature abundance. Although our results indicate the importance of the type of use over the type of container, we encourage the use of both classification criteria for artificial breeding sites of mosquitoes, mainly because these are complementary. Additionally, generalized linear mixed models allowed us to analyse predictor variables at different scales (container/house/block) and consider the lack of independence between observations. An exhaustive analysis of artificial breeding sites that use this analytical methodology can lead to new information that could help designing more appropriate tools for dengue surveillance and control.

## Background

*Aedes aegypti* is the principal vector of dengue virus and is also a vector of Zika, chikungunya, and urban yellow fever. This mosquito is distributed between 35° North and 35° South, although recent works predict expansion in its distribution [1, 2]. Houses and their surroundings within urban areas provide this mosquito with food, shelter, reproduction, oviposition and development sites. Breeding sites are usually artificial containers that accumulate water, e.g. bottles, buckets, tanks, and tires. Since 2013, the viruses transmitted by *Ae. aegypti* have shown an important expansion, particularly in Latin America [3–5]. In the absence of specific antiviral therapies or globally accepted vaccines [6], dengue control depends on measures in the different stages of the vector. Thus, artificial breeding sites are an excellent target for the control of urban mosquitoes.

Despite being widely studied, two important difficulties can be identified in the studies on artificial breeding sites. Firstly, there is no consensus on the classification of the containers. Most authors focus on the type of container, such as buckets, bottles, vases, tanks, generating an extensive list of container types [7–12]. Other authors use several criteria simultaneously, combining type, material, and capacity of containers [10, 13]. However, none of these classifications contemplate that a particular container could have different attractiveness for female mosquitoes, depending on functional characteristics determined by human actions. For example, the accumulation of water and/or organic matter could be favoured by the type of use or location of the container. According to this, another approach is based on the use that containers receive, such as water storage, household chores, etc. [14–18]. The absence of a unifying framework for the classification of containers makes the comparison between studies difficult and may affect decision making in control measures.

Secondly, an important limitation is identified regarding the statistical analyses. Most studies of abundance or presence of immatures in containers ignore the hierarchical structure of the data, i.e., containers nested in houses and houses nested in blocks. Typically, data from containers belonging to the same house are considered independent when they are not [8–10, 13, 14, 17, 18]. Ignoring this lack of independence could lead to a wrong statistical inference, over- or underestimating coefficients and *P*-values [19, 20]. Generalized linear mixed models (GLMM) are a flexible and powerful analytical tool, widely used to analyse aggregated data by including random effects, allowing an estimation of the variability explained by the nesting factors [20–22]. Although the implementation of these models has been growing in recent years, studies on urban mosquitoes breeding sites using these models are still scarce, mainly limited to presence studies [23–26].

The present study proposed a framework for the study of artificial breeding sites of *Ae. aegypti* in houses in the Colón city (Entre Ríos, Argentina) as an example. To achieve this, we: 1) classified the containers according to different criteria based on physical, functional and location characteristics, and compared the information provided between the criteria ‘type of container’ and ‘type of use’ of the container, and 2) proposed a methodology for statistical analysis of abundance data based on GLMM, in which the lack of independence between the containers belonging to the same house and/or the same block was contemplated.

## Methods

### Study area

This work was carried out in Colón city (32° 13′00″S; 58° 08′00″W, Entre Ríos, Argentina, 57 m above sea level). This city is located on the shore of the Uruguay River and is characterized by mild weather with an annual average temperature of 17 °C and annual mean precipitation of 1000 mm. The wet season runs from October to April, with an average maximum rainfall reaching 130 mm in March (National Weather Service).

This city has more than 62 000 inhabitants (National Institute of Statistics and Census, 2010) distributed in 520 blocks, and a satisfactory household water supply, sanitation facilities, electricity services, and rubbish collection. Colón is a tourist city, mainly occupied by residences, shops, and hotels, and shows different urbanization levels, from densely built areas (houses, shops, sidewalks, etc.) up to barely built ones (few houses, absence of shops and dirt roads). The city has 7.6% of households with unmet basic needs, slightly less than the national total (9.1%).

Colón is near to important land and fluvial routes that connect with dengue-endemic areas, i.e., northern Argentina and bordering countries (Brazil, Paraguay, Bolivia, and Uruguay). During the last two major dengue outbreaks in Argentina in 2009 and 2016 (26 000 and 76 000 confirmed cases) [27, 28], autochthonous cases were detected in neighbouring cities of Colón. Simultaneously to the dengue epidemic of 2016, occurred co-circulation of the chikungunya and Zika viruses [28]. This epidemiological context places Colón as a city of interest to carry out studies related to *Ae. aegypti.*

### Sampling design and entomological survey

**Fig. 1:**
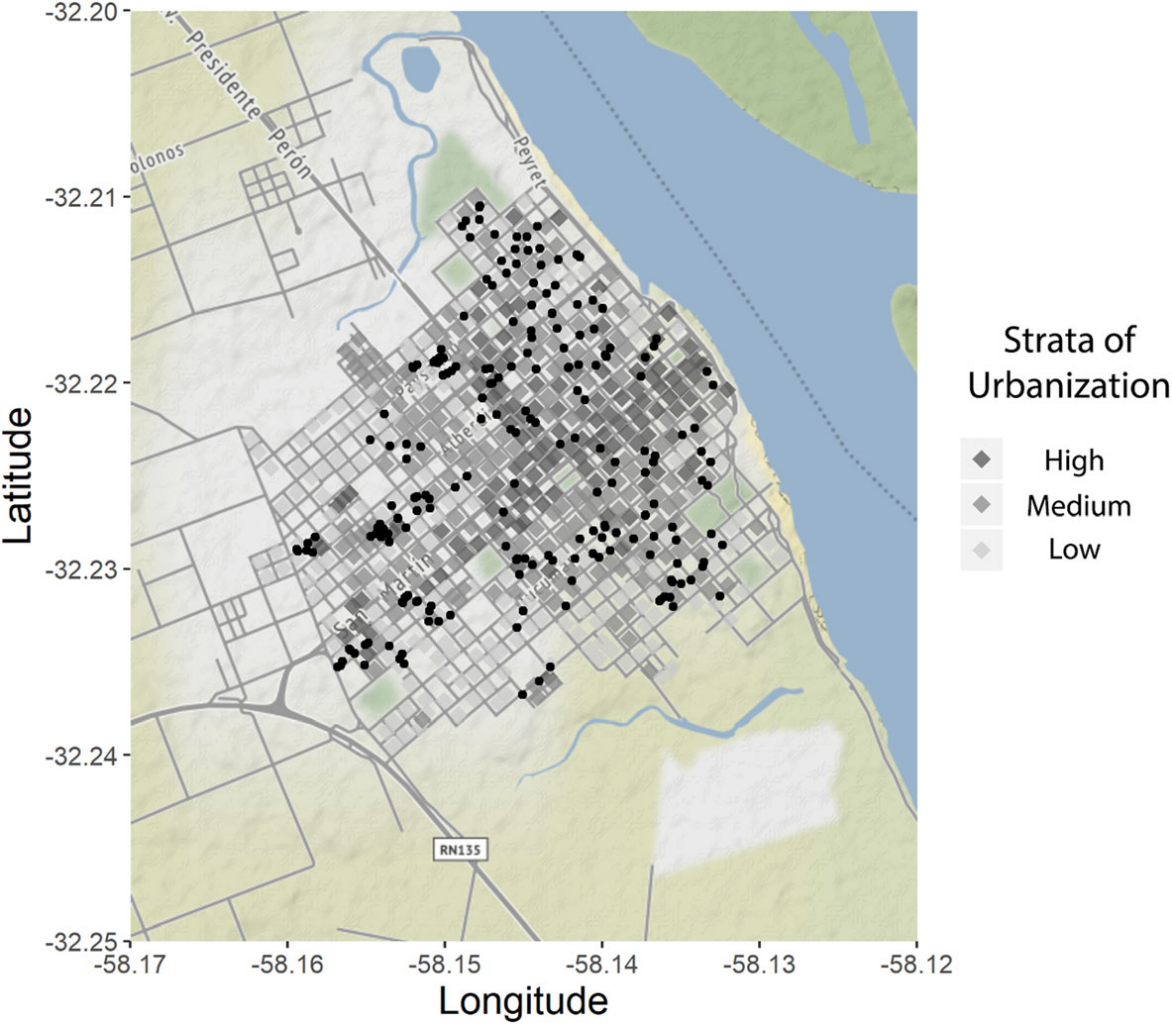
Map of the city of Colón, Entre Ríos, with the degrees of urbanization in which the city was stratified in greyscale. The black points are the houses that were visited during the study

A cross-sectional study was carried out between March and April 2014 when the *Ae. aegypti* abundance in the region is higher [29, 30]. Given the heterogeneity of this city, we employed a stratified two-stage sampling design. The city was stratified according to the degree of urbanization of each block (Fig. 1). We determined the percentage of built-up area for each city block based on satellite photographs provided by Google Earth™ (date: 03/22/2014). The degree of urbanization ranged between 20 and 78%, and three strata were defined: low (< 45% of built-up area, *n* = 201 blocks), medium (≥ 45% and ≤ 55% of built-up area, *n* = 193 blocks) and high urbanization (> 55% built-up area, *n* = 125 blocks).

From each stratum, between 20 and 25% of blocks were randomly selected (low = 52, medium = 42 and high = 30) and then, one or two houses from each block (*n* = 207 houses). All containers with water located outdoors were inspected to record the presence of immature mosquitoes. Containers with at least one immature mosquito were considered as positive containers (breeding site). The water was filtered through a 350 μm mesh network and in the containers with more than 25 l, the network was passed three to six times, taking samples of the surface layer (30 cm depth). In these cases, the abundance was referred to the volume of water contained in the superficial layer of the container. Larvae were fixed in 70% ethanol in situ. Pupae were maintained alive for the identification of adults. All individuals were identified using the Darsie key [31]. Only larvae in stages three and four were identified.

### Container classification

Water containers were classified according to seven criteria clustered in three dimensions: a) Physical: Type of Container, Material, Opening Surface, b) Functional: Type of Use, Use Status (in use or not at the time of the survey), and c) Location: Sunlight Exposure, and Coverage by Roof. Type of Container was defined in 11 categories: animal water dishes, bottles, buckets, cans, flower pots, jars (plastic containers with a capacity of fewer than 3 l), kitchen items (bowls, saucepans, pans, etc.), swimming pools, tanks, tires, and others (wheelbarrows, water sinks, boats, etc.). Type of Use was defined in eight categories according to the use assigned by the house owners: Construction and Spare Parts Elements (CSPE), as buckets, cans, tires, etc.; Household Chores, mainly buckets used for cleaning, laundering, garbage, etc.; Gardening, as pots, flower pot holders, containers used for watering, rooting plants, etc.; Pets Items, as water dishes, food bowls, toys; Returnable Bottles, as plastic or glass returnable containers; Water Storage, as drums, tanks, drinking water drums or buckets; Others, as a heterogeneous category of different uses as ornaments, awnings, swimming pools, etc.; and Useless Objects, considered as waste by the house inhabitants. Container Opening Surface was classified in: Small < 20 cm^2^, Medium ≥20 cm^2^ and < 300 cm^2^, Large ≥300 cm^2^ and < 1000 cm^2^ and Very Large ≥1000 cm^2^. Material was classified in five categories: Clay (as brick and concrete), Glass, Metal, Plastic and Rubber. Sunlight Exposure was classified as Shadow (less than 20% of sunlight exposure) or Sun (20% of sunlight exposure or more), according to Wong et al. [32]. Coverage by Roof was classified as Yes or No.

### Data analysis

Different indexes were estimated by container classification category [12, 33], for each house with water containers and breeding sites. To consider the clustering of the containers of the same house, we averaged the indexes among those houses that presented containers with water and breeding sites. The index of available containers (IAC) was estimated as the proportion of containers from each category divided by the total of containers in houses containing at least one water container. The index of contribution to breeding sites (ICBS) was estimated as the proportion of positive containers from each category divided by the total of positive containers in houses with positive containers. The breeding preference ratio (BPR) was estimated as the ratio between IBS and IAC for each category. Values lower than one would indicate that this category was not attractive for female mosquitoes, whereas values higher than one would indicate that this category was exploited. Values close to one would suggest that the container category was used in the same proportion it was available.

To identify the container characteristics associated with the abundance of *Ae. aegypti* immatures*,* zero-inflated GLMM were used with a negative binomial distribution and log link [34]. To take into account the nested design, we included Blocks and Houses as variables of random effects into the models. To evaluate the fit of the models, we used DHARMa package [35]. To avoid the redundancy between the variables Type of Container and Type of Use, we analysed each variable separately (Model 1 and Model 2), but including all the remaining independent variables. The liquid volume of the container was log-transformed before being included in the models. The analytical sample only included houses that had containers with water (*n* = 149).

Comparisons between categories for qualitative variables were made using the Dunnett test. For Type of Container and Type of Use, the category ‘Others’ was used as a reference because due to its high heterogeneity it could be considered as a neutral category; for Container Opening Surface, Small was used as a reference.

Analyses were carried out using glmmTMB [36] and emmeans packages [37] in R version 3.3.1 (R Core Team, R Foundation for Statistical Computin, Vienna, Austria 2019) [38]. Likelihood ratio test was used to evaluate the random variables using the anova function of stats package [38].

## Results

### General results

A total of 522 water containers were inspected in 207 houses, and 136 of the containers had immature mosquitoes (7336 *Ae. aegypti* and 1977 *Culex pipiens* complex). *Ae. aegypti* was found in 131 containers, meanwhile, immatures of *Culex pipiens* complex were found in 33 containers, and they coexisted in 28 containers. Table S1 summarizes the number of immature mosquitoes per container, discriminated by stage. From sampled houses, 58 did not have water containers, 75 had water containers without immatures of *Ae. aegypti*, and 74 were breeding sites of *Ae. aegypti*. The average number of *Ae. aegypti* per house was 19.7 (Standard Deviation = 48.4, median = 0, range: 0–465).

Epidemiologic indexes by physical, functional and use characteristics of containers are shown in Table 1. IAC and ICBS showed similar patterns, as well as BPR and the average abundance per house of *Ae. aegypti* immatures.

**Table 1:** Epidemiologic indexes by container classification

Characteristics	Categories	Total number of Water Containers	Total number of Breeding Sites	IAC (mean by house)	ICBS (mean by house)	BPR (mean by house)	Abundance of Immatures by house (mean)
Physical	Type of Container						
	Animal Water Dishes	43	4	0.1	0.03	0.3	0.7
	Bottles	120	6	0.1	0.03	0.3	0.1
	Buckets	139	43	0.3	0.4	1.3	28.1
	Cans	34	15	0.1	0.1	1.2	7.1
	Flower Pots	16	4	0.02	0.02	1.0	15.8
	Jars	52	11	0.1	0.1	0.8	18.1
	Kitchen Items	34	11	0.1	0.1	1.1	10.5
	Swimming Pools	17	7	0.05	0.05	1.0	13.0
	Tanks	13	8	0.04	0.1	2.0	38.1
	Tires	23	15	0.05	0.1	1.4	55.1
	Others	31	7	0.1	0.1	1.1	24.9
	Material						
	Clay	12	3	0.03	0.02	0.7	13.7
	Glass	122	9	0.2	0.1	0.5	2.0
	Metal	85	28	0.2	0.2	1.0	15.2
	Plastic	271	72	0.6	0.6	1.0	22.3
	Rubber	32	19	0.1	0.1	1.6	47.9
	Opening Surface						
	Small	127	7	0.1	0.04	0.4	0.3
	Medium	200	61	0.5	0.5	1.1	20.7
	Large	162	49	0.3	0.3	1.0	17.7
	Very Large	33	14	0.1	0.1	1.3	30.2
Functional	Type of Use						
	CSPE	55	23	0.1	0.1	1.4	22.2
	Household Chores	45	13	0.1	0.1	1.2	27.9
	Gardening	57	19	0.1	0.2	1.1	37.4
	Pets Items	109	10	0.3	0.1	0.4	0.6
	Returnable Bottles	91	5	0.05	0.02	0.4	0.1
	Water Storage	36	11	0.1	0.1	1.3	24.1
	Others	33	9	0.1	0.1	0.9	9.0
	Useless Objects	96	41	0.2	0.3	1.6	37.4
	Status of Use						
	Out of Use	294	94	0.5	0.7	1.4	27.7
	In Use	228	37	0.5	0.4	0.7	14.6
Location	Sunlight Exposure						
	Shadow	207	58	0.4	0.4	1.0	21.3
	Sun	315	73	0.6	0.6	1.0	16.3
	Coverage by Roof						
	Yes	84	15	0.2	0.1	0.6	3.6
	No	438	116	0.8	0.9	1.1	22.4

*CSPE* Construction and spare parts elements, *IAC* Index of available containers, *ICBS* Index of contribution to breeding sites, *BPR* Breeding preference ratio

**Fig. 2:**
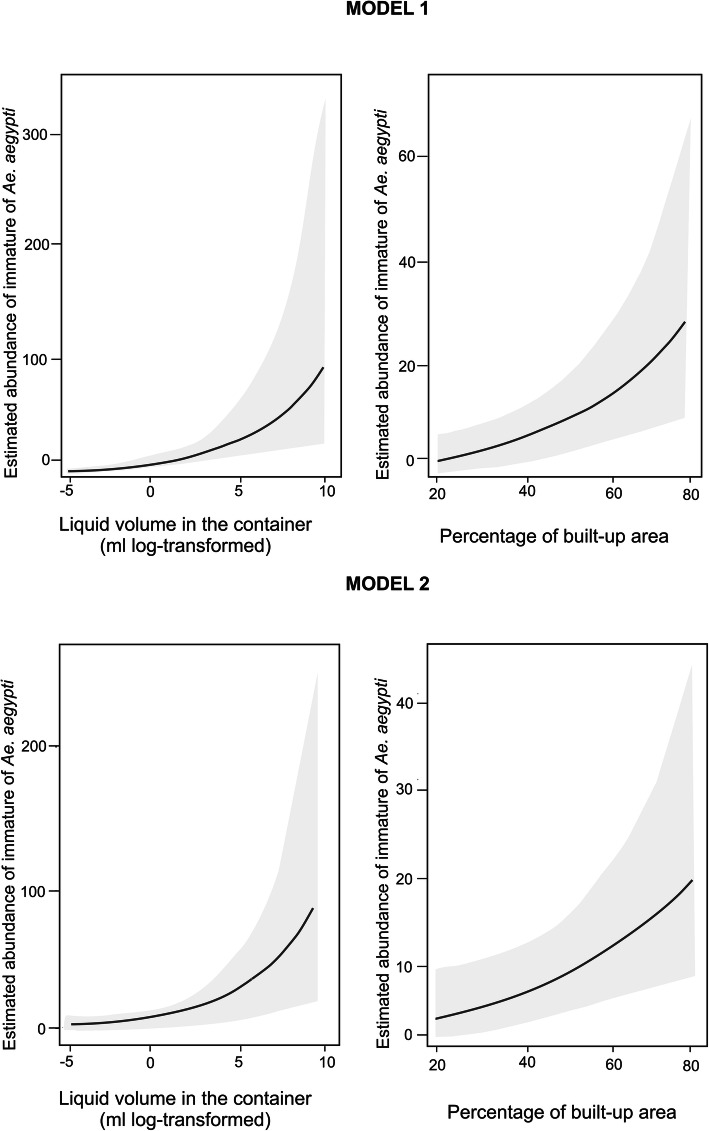
Relationship between abundance of *Aedes aegypti* immatures and liquid volume in the container (left) or the percentage of built-up area in the block (right), by Model 1 and 2. 95% confidence interval is shown

The final models (Models 1 and 2) presented coincidences and differences in the variables associated with the abundance of immatures (Table 2). Status of Use, coverage by roof, liquid volume and percentage of built-up area were significantly associated with immatures abundance in both models (*P* < 0.05) (Fig. 2), while material was not significantly associated with abundance in neither of them (*P* > 0.05). Out of Use and No-Coverage by Roof showed a higher abundance of immatures compared with the categories Out of Use and Yes-Coverage by Roof, respectively. Additionally, the liquid volume and the percentage of the built-up area showed positive associations with abundance (*P* < 0.01).

**Table 2:** Abundance of *Aedes aegypti* immatures estimated by generalized linear mixed models. The significance values for the categorical variables correspond to the Dunnet test

**Model 1**			
**Categories**	**Estimated Abundance (CI 95%)**	**Categories**	**Estimated Abundance (CI 95%)**
Type of Container		Status of Use	
Others (Reference)	15.26 (5.13-45.40)	In Use (Reference)	7.24 (3.17-16.60)
Animal Water Dishes	4.17 (1.02-17.10)	Out of Use	17.14 (8.74-33.60) *
Bottles	0.62 (0.18-2.10) ***	Coverage by Roof	
Buckets	27.80 (12.62-61.30)	Yes (Reference)	7.02 (2.72-18.10)
Cans	16.56 (7.52-36.50)	No	17.70 (10.25-30.50) *
Flower Pots	55.53 (17.64-174.80)	Sunlight Exposure	
Jars	15.07 (4.93-46.10)	Sun (Reference)	8.40 (3.63-19.40)
Kitchen Items	9.02 (3.04-26.70)	Shadow	14.80 (8.05-27.02) *
Swimming Pools	5.39 (1.34-21.60)	Volume (log litres) **	
Tanks	21.71 (7.19-65.60)	Built-up area **	
Tires	20.46 (7.77-53.90)		
**Model 2**			
**Categories**	**Estimated Abundance (CI 95%)**	**Categories**	**Estimated Abundance (CI 95%)**
Type of Use		Opening Surface	
Others (Reference)	2.21 (0.58-8.39)	Small (Reference)	0.72 (0.15-3.54)
CSPE	9.52 (3.54-25.59) *	Medium	17.71 (9.23-34.00) ***
Household Chores	13.01 (5.24-32.30) *	Large	10.82 (4.67-25.07) **
Gardening	15.73 (7.17-34.51) *	Very Large	12.79 (5.09-32.09) **
Pets Items	1.48 (0.50-4.38)	Coverage by Roof	
Returnable Bottles	5.21 (0.93-29.15)	Yes (Reference)	4.22 (1.67-10.06)
Useless Objects	7.40 (3.09-17.73)	No	10.01 (5.69-17.06) *
Water Storage	12.96 (4.28-39.29) *	Volume (log litres) **	
Status of Use		Built-up area **	
In Use (Reference)	3.43 (1.31-8.94)		
Out of Use	12.31 (6.72-22.55) **		

*CSPE* Construction and Spare Parts Elements, *CI* Confidence interval *** *p* < 0.001; ** *p* < 0.01; * *p* < 0.05

Model 1 showed significant differences in Type of Container and Sunlight Exposure (*P* < 0.05). Bottles showed a lower number of immatures than the reference category, while Shadow showed a higher abundance of immatures concerning Sun. In model 2_,_ Type of Use, Coverage by Roof and Container Opening Surface were significantly associated with immatures abundance (*P* < 0.05). Household Chores, Gardening, Water Storage, and CSPE showed a higher abundance of immatures compared to the reference category. For Opening Surface, the categories Medium, Large and Very Large showed more immatures abundance than Small (Table 2). In both models, Blocks and Houses explained a significant variability (*P* < 0.05).

## Discussion

In the present study, we identify characteristics of breeding sites of *Ae. aegypti* in an urban area of epidemiologic relevance. We used an exhaustive description of water containers applying an appropriate statistical analysis.

Regarding the container’s physical characteristics, we found an increase in immatures abundance as the liquid content increases, coinciding with other studies [7, 14, 23, 39]. Large volumes of water could provide more food and habitat stability than small volumes. However, other works have not found a relationship between volume and presence or abundance of this mosquito [8, 24, 40].

Bottles were the most frequent container type but the least used as breeding sites, showing a low abundance of immatures, coinciding with other studies [12, 16, 41]. The small opening surfaces could result in a less detectable container. Besides, the bottles found are usually glass with a smooth surface that may difficult the oviposition. This is consistent with the BPR estimates for glass and small opening containers.

Although buckets did not show a significantly higher abundance of immatures than the reference category, they were the most frequent breeding sites, showing considerable exploitation. Similar results were found in other studies [16, 41, 42]. These high values could be related to the capacity of the bucket to contain considerable volumes of water, to the medium opening surfaces and aspects related to its uses associated with the accumulation of water (gardening and housework).

The positive association between the abundance of immatures and opening surfaces greater than 20 cm^2^ coincided with previous works [23, 32]. Furthermore, we detected a high proportion of breeding sites in containers of medium and large surfaces, and greater preference for those with large opening surfaces, as reported in other works [18, 43, 44]. Containers of medium and large opening surface, between 20 cm^2^ and 5000 cm^2^ (buckets up to tanks or drums), could be attractive for females due to their great detectability and easy access. Moreover, a large opening surface is generally associated with larger volumes of liquid that can provide more stable environments than those with small volumes.

Regarding the function of the container, we detected a high number of immatures in containers that were out of use and in those related to household chores, gardening, water storage, construction, and spare parts. This trend was consistent with the values of the eco-epidemiological indexes and previous studies [16, 17]. Useless containers or those related to gardening activities, such as containers with rooting plants and flower pot holders, involve the presence of generally organic material from the soil, remainders of vegetables or insects, which allow the development of bacteria that constitute food for larvae [45].

Elements for construction, household chores and spare parts, represented mainly by buckets, could share a similar use dynamic, apart from having the attractive characteristics already mentioned for buckets. Although these containers are often used, they usually contain small volumes of water from previous usages (transportation of liquids, cleaning, etc.). Fluctuations in the water level can cause the hatching of eggs. Although it is expected that the next use will eliminate a large number of immatures by removing water, the number of eggs deposited could be greater than the number of immatures removed during the peak of abundance. This dynamic, together with a low frequency of use would provide enough time to maintain the number of immatures observed. We also detected higher abundance in out-of-use containers than in those in use. This is consistent with previous works that found that a low frequency of use (every seven days or more) was positively associated with the presence or abundance of immatures of *Ae. aegypti* and other species [25, 26, 46]. Although the containers used for water storage were not frequent breeding sites, they were positively associated with the abundance of immatures, agreeing with other studies [9, 11–13, 24, 25, 46, 47]. Generally, this kind of container has large opening surfaces, capacity to contain large volumes of water, are not daily used and are not usually emptied [11, 12]. Although Hammond et al. [46] detected that water storage containers are used at least once a week, in those cases where the containers were not used for more than four days, they found a positive association with the presence of immatures. A low frequency of use together with an incomplete water drainage can provide a suitable environment for immature development.

Concerning the location of the containers, we detected that containers that were not under roof coverage had a higher tendency to be breeding sites and to have more immatures than those under roof coverage, unlike other authors [48, 49]. The absence of a roof would favour the accumulation of rainwater and organic material, thus facilitating the oviposition and development of mosquito larvae. Although shaded containers had a greater abundance of immature compared to sunny ones, both conditions showed BRP values close to one, suggesting that there was no preferential use for the condition. Previous studies showed that *Ae. aegypti* females prefer containers in the shadow [18, 24, 25, 39, 49, 50]. However, other studies did not find a relationship between abundance or presence of immatures with levels of exposure to sunlight [48, 51, 52] or found an opposite relationship [32]. The positive association between the abundance of immatures and shaded containers might be because the shadow could protect immatures from high temperatures and evaporation, increasing their survival.

At the block level, the positive relationship between the degree of urbanization and the abundance of immatures found is consistent with previous studies [47, 49, 53, 54]. This relationship could be explained by a greater availability of containers and hosts in more populated areas. Contradicting this, Carbajo et al. [55] found the lowest abundance of *Ae. aegypti* in the most urbanized and highly populated areas of Buenos Aires city, where the free flight of mosquitoes to zones with vegetation and humidity could be limited. However, Colón city is not characterized by having fully built-up areas with high buildings as is Buenos Aires city. From our results, houses and blocks were a significant source of variation in immatures abundance, highlighting the importance of their inclusion in the model. Households can give information about the particular dynamic of their containers (e.g. predisposition to accumulate a specific type of container, frequency of cleaning and home maintenance), while on a higher scale, blocks can indicate the dynamic per hectare (proportion of houses and inhabitants, levels of vegetation, frequency of rubbish collection by the municipality, etc.). These different scales of information could help to understand how the abundance of *Ae. aegypti* immatures are regulated.

### Advantages and drawbacks in the categorization according to type and use of container

The characterization of the containers according to their use allows detecting more general characteristics of the breeding sites related to home activities or practices. Consequently, recommendations can be made to the communities about the care of some activities at home, such as gardening and domestic chores. The study of the type of container allows the identification of those particular objects that would be acting as the most important breeding sites. This information could make them more easily recognizable by the general population and the staff of government health agencies that are in charge of developing preventive measures to control these mosquitoes. However, these classifications are not often implemented together in the same study, with a few exceptions [14–16]. Compartmentalized approaches would be incomplete, and even incorrect, because the same type of object can have very different dynamics. For example, we found that buckets, which were ones of the most infested and exploited containers, were associated with quite different uses. Therefore, the recommendations to avoid having artificial breeding sites of urban mosquitoes should be specific to the use instead of encompassing them only as ‘caring of buckets’, where their use is not taken into account.

Another advantage of classifying containers by their use is that it allows a lesser number of categories to analyse in comparison to the container type classification, which contributes to a greater power in statistical analyses than comparisons with extensive classification and poorly representative categories.

Also, the classification of containers according to their use allows comparing studies across countries. The types of artificial containers can vary between places depending on the economic resources, cultural aspects, typical materials of the region and their availability, among other aspects. Some categories may not exist in some contexts, such as ships or coconut shells, making it difficult and sometimes impossible to compare and generalize results. A complementary classification with fewer categories, can provide useful elements to discuss more generally among cities or regions around the world.

Although it could seem that the implementation of the type of use as a criterion would be more appropriate than the classification by type of container, we consider that both approaches are valid and important. Depending on the goals of the study (conclusions on a regional vs global scale), one approach may be more practical than the other. However, since both approaches provide complementary information, they should be used simultaneously, going from a more general approach to a more detailed one. This could allow a more comprehensive standpoint for recommendations on the key containers linked to the abundance of *Ae. aegypti* immatures.

### Limitations of the study

First, only a single sampling was carried out, precluding the analysis of temporal variations. Nevertheless, the study allowed the characterization of breeding sites in the moment of the highest risk of transmission of several arboviruses. Second, we did not take into account potential containers (e.g., empty containers but exposed to the rain). Future longitudinal studies could provide information about the dynamics of these containers, and on the intra- and interannual variations in the abundance of *Ae. aegypti* immatures. Third, our conclusions are limited to containers from houses, because the sampling did not include public space nor private property beyond the houses. Although we are aware that these spaces also could have artificial containers that can function as breeding sites, our goal was to evaluate the type of use that the containers receive by the inhabitants of the houses.

## Conclusions

The present study allowed us to identify artificial breeding sites associated with the abundance of *Ae. aegypti* inmatures in urban areas. We detected that the containers related to the accumulation of water or out of use are strongly associated with the number of immatures. Besides, we found that immatures abundance was positively associated with containers with increasing opening surface and volume, without roof cover and exposed to shadow.

Additionally, we consider that the present work presents some novel contributions. First, it is one of the few works studying the abundance of *Ae. aegypti* immatures in urban breeding sites based on broad criteria container classification using hierarchical models. We must emphasize that these results were obtained through a more adequate statistical analysis than those frequently implemented in studies on this topic. This methodology allowed us to obtain more reliable estimates for the variables of interest, and evaluate the importance of variables of higher spatial hierarchy such as dwellings or blocks. Second, we discussed the benefits and disadvantages of the different criteria for classifying artificial containers. Although the classifications of containers used here are not exempt from biases, we consider that these types of studies allow us to highlight discussions and analysis methodologies that enrich the understanding of complex issues. This is especially important in fields that are closely related to the management of vector control strategies since it can provide novel surveillance or control approaches.

## Supplementary information

**Additional file 1: Table S1.** Number of immatures discriminated by stage and number of adults emerged from the pupae discriminated by sex.

## Data Availability

The datasets used and/or analysed during the current study are available from the corresponding author on reasonable request.

## Abbreviations

GLMM: Generalized Linear Mixed Models
CSPE: Construction and Spare Parts Elements
IAC: Index of Available Containers
ICBS: Index of Contribution to Breeding Sites
BPR: Breeding Preference Ratio
CI: Confidence interval
