# Supplementary material for: Different approaches to characterize artificial breeding sites of Aedes aegypti using generalized linear mixed models
Source: Infect Dis Poverty. 2020 Jul 31;9:107. doi: 10.1186/s40249-020-00705-3 (PMC7393697; doi:10.1186/s40249-020-00705-3)
Supplement: Supplementary file 1 — Additional file 1: Table S1. Number of immatures discriminated by stage and number of adults emerged from the pupae discriminated by sex. [file 40249_2020_705_MOESM1_ESM.docx]

Table S1. Number of immatures discriminated by stage and number of adults emerged from the pupae discriminated by sex.

|  |  | ***Ae. aegypti*** | | | ***Cx. pipiens complex*** | | |
| --- | --- | --- | --- | --- | --- | --- | --- |
| **Characteristics** | **Categories** | **Larvae (Stage 3-4)** | **Pupae** | **Adults (♂ ; ♀)** | **Larvae (Stage 3-4)** | **Pupae** | **Adults (♂ ; ♀)** |
| Physical | **Type of Container** |  |  |  |  |  |  |
|  | Animal Water Dishes | 36 | 0 | (0;0) | 40 | 0 | (0;0) |
|  | Bottles | 21 | 1 | (1;0) | 0 | 0 | (0;0) |
|  | Buckets | 2997 | 235 | (131;104) | 667 | 113 | (61;52) |
|  | Cans | 324 | 25 | (15;10) | 178 | 4 | (2;2) |
|  | Flower Pots | 399 | 14 | (6;8) | 9 | 2 | (1;1) |
|  | Jars | 735 | 59 | (31;28) | 115 | 5 | (2;3) |
|  | Kitchen Items | 288 | 18 | (7;11) | 8 | 0 | (0;0) |
|  | Swimming Pools | 194 | 14 | (8;6) | 122 | 5 | (3;2) |
|  | Tanks | 431 | 38 | (20;18) | 84 | 0 | (0;0) |
|  | Tires | 825 | 38 | (26;12) | 269 | 0 | (0;0) |
|  | Others | 647 | 6 | (4;2) | 345 | 11 | (7;4) |
|  |  |  |  |  |  |  |  |
|  | **Material** |  |  |  |  |  |  |
|  | Clay | 162 | 2 | (1;1) | 0 | 0 | (0;0) |
|  | Glass | 283 | 8 | (6;2) | 0 | 0 | (0;0) |
|  | Metal | 1256 | 36 | (18;18) | 579 | 17 | (10;7) |
|  | Plastic | 4182 | 352 | (191;161) | 919 | 123 | (66;57) |
|  | Rubber | 1014 | 50 | (33;17) | 339 | 0 | (0;0) |
|  |  |  |  |  |  |  |  |
|  | **Opening Surface** |  |  |  |  |  |  |
|  | Small | 29 | 5 | (4;1) | 0 | 0 | (0;0) |
|  | Medium | 3452 | 213 | (111;102) | 704 | 24 | (13;11) |
|  | Large | 2504 | 205 | (121;84) | 439 | 37 | (20;17) |
|  | Very Large | 912 | 25 | (13;12) | 694 | 79 | (43;36) |
|  |  |  |  |  |  |  |  |
| Functional | **Type of Use** |  |  |  |  |  |  |
|  | CSPE | 819 | 19 | (11;8) | 303 | 0 | (0;0) |
|  | Domestic Chores | 1154 | 109 | (60;49) | 601 | 76 | (41;35) |
|  | Gardening | 1711 | 88 | (44;44) | 572 | 55 | (30;25) |
|  | Pets Items | 75 | 9 | (6;3) | 48 | 0 | (0;0) |
|  | Returnable Bottles | 16 | 1 | (1;0) | 0 | 0 | (0;0) |
|  | Water Storage | 549 | 16 | (9;7) | 20 | 0 | (0;0) |
|  | Others | 222 | 17 | (11;6) | 88 | 4 | (2;2) |
|  | Useless Objects | 2351 | 189 | (107;82) | 205 | 5 | (3;2) |
|  |  |  |  |  |  |  |  |
|  | **Status of Use** |  |  |  |  |  |  |
|  | Out of Use | 5009 | 365 | (205;160) | 1225 | 86 | (47;39) |
|  | In Use | 1888 | 83 | (44;39) | 612 | 54 | (29;25) |
|  |  |  |  |  |  |  |  |
| Location | **Sunlight Exposure** |  |  |  |  |  |  |
|  | Shadow | 2978 | 125 | (64;61) | 694 | 53 | (29;24) |
|  | Sun | 3919 | 323 | (185;138) | 1143 | 87 | (47;40) |
|  |  |  |  |  |  |  |  |
|  | **Coverage by Roof** |  |  |  |  |  |  |
|  | Yes | 273 | 18 | (9;9) | 67 | 10 | (5;5) |
|  | No | 6624 | 430 | (240;190) | 1770 | 130 | (71;59) |
|  | **Total** | 7345 | | (249;199) | 1977 | | (76;64) |

CSPE = Construction and Spare Parts Elements.
